# Hipertensão na Gravidez: Das Diretrizes à Prática do Cardiologista

**DOI:** 10.36660/abc.20260047

**Published:** 2026-08-14

**Authors:** Walkiria Samuel Ávila

**Affiliations:** 1 Hospital das Clínicas da Faculdade de Medicina da Universidade de São Paulo Instituto do Coração São Paulo SP Brasil Instituto do Coração do Hospital das Clínicas da Faculdade de Medicina da Universidade de São Paulo, São Paulo, SP – Brasil

**Keywords:** Hipertensão, Gravidez, Pré-Eclâmpsia, Guia de Prática Clínica, Fatores de Risco de Doenças Cardíacas

## Abstract

**Figure:**
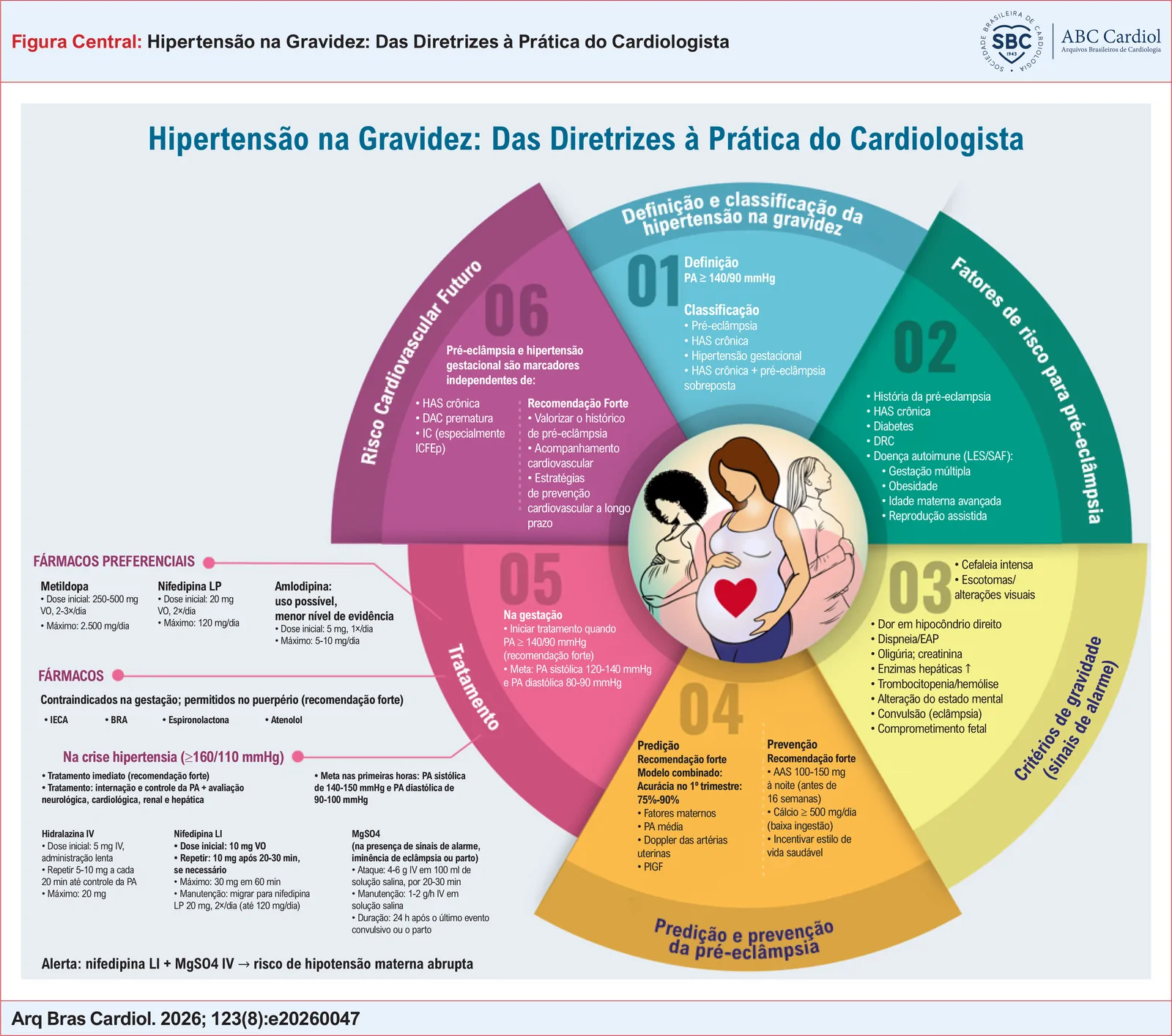
AAS: ácido acetilsalicílico; BRA: bloqueador dos receptores da angiotensina II; DAC: doença arterial coronariana; DRC: doença renal crônica; EAP: edema agudo de pulmão; HAS: hipertensão arterial sistêmica; IC: insuficiência cardíaca; ICFEp: IC com fração de ejeção preservada; IECA: inibidor da enzima conversora da angiotensina; IV: intravenoso; LES: lúpus eritematoso sistêmico; LI: liberação imediata; LP: liberação prolongada; PA: pressão arterial; PlGF: fator de crescimento placentário; SAF: síndrome antifosfolípide; VO: via oral.

## Introdução

A Diretriz Brasileira de Hipertensão Arterial – 2025^1^ da Sociedade Brasileira de Cardiologia aborda de forma objetiva a hipertensão na gestação, dedicando um capítulo específico ao tema. O documento contempla definições diagnósticas, estratégias de predição e profilaxia da pré-eclâmpsia, manejo terapêutico durante a gestação e o puerpério, além da avaliação do risco cardiovascular futuro associado às doenças hipertensivas da gravidez.

No cenário internacional, as *2025 ESC Guidelines for the Management of Cardiovascular Disease and Pregnancy*, apresentam algoritmos detalhados para a avaliação da proteinúria, o manejo de emergências e o tratamento da hipertensão e da pré-eclâmpsia. Suas recomendações baseiam-se predominantemente em consenso de especialistas, refletindo a escassez de ensaios clínicos randomizados nessa área.^2^

Embora adotem metodologias distintas — a diretriz brasileira fundamentada no sistema *Grading of Recommendations Assessment, Development and Evaluation* e a europeia estruturada em classes de recomendação e níveis de evidência —, ambos os documentos convergem em aspectos centrais da prática clínica. Entre eles, destacam-se o reconhecimento da pressão arterial (PA) ≥ 140/90 mmHg como critério diagnóstico de hipertensão, a recomendação de tratamento para formas leves a moderadas da doença, a necessidade de intervenção imediata diante da hipertensão grave e a importância do acompanhamento cardiovascular contínuo de mulheres com histórico de doença hipertensiva da gravidez. Essa convergência reflete o alinhamento progressivo das principais diretrizes internacionais após a publicação das recomendações da International Society for the Study of Hypertension in Pregnancy em 2021, que padronizaram critérios diagnósticos e métodos de aferição da PA, além de reforçarem recomendações favoráveis ao tratamento ativo da hipertensão durante a gestação.^3,4^ Posteriormente, essas recomendações foram fortalecidas pelos resultados do estudo CHAP.^5^

Apesar da crescente uniformização das recomendações, a aplicação prática desse conhecimento ainda representa um desafio para o cardiologista generalista. A classificação das síndromes hipertensivas da gestação nem sempre é intuitiva, e situações frequentes (p.ex., hipertensão grave, elevação pressórica no puerpério e pré-eclâmpsia sobreposta) exigem reconhecimento precoce e tomada de decisão rápida. Nesse contexto, ferramentas de síntese clínica podem auxiliar a aplicação das recomendações atuais à prática assistencial. A Figura Central deve ser interpretada de forma sequencial, acompanhando os módulos numerados que sintetizam os principais aspectos da hipertensão na gravidez, desde a definição diagnóstica e a estratificação de risco até o manejo clínico e as implicações cardiovasculares de longo prazo.

### Definição, classificação e critérios de gravidade

Considera-se hipertensão na gravidez a presença de PA ≥ 140/90 mmHg, valor que constitui o limiar diagnóstico comum às diferentes formas de doença hipertensiva da gestação.^1–4^ A correta distinção entre essas entidades representa o primeiro passo para uma abordagem segura, uma vez que orienta a intensidade da vigilância clínica, a urgência terapêutica e o planejamento obstétrico (Figura Central).

Na prática clínica, algumas apresentações configuram formas graves da doença hipertensiva da gestação e exigem reconhecimento imediato. Entre elas destacam-se hipertensão grave (PA sistólica ≥ 160 mmHg e/ou PA diastólica ≥ 110 mmHg), eclâmpsia (convulsões tônico-clônicas generalizadas), síndrome HELLP (hemólise, elevação de enzimas hepáticas e trombocitopenia) e síndrome da encefalopatia posterior reversível, caracterizada por manifestações neurológicas associadas a edema vasogênico predominantemente parieto-occipital. Essas condições refletem comprometimento agudo de órgãos-alvo e marcam um importante ponto de inflexão prognóstico, demandando intensificação imediata do cuidado, implementação de terapias específicas e avaliação do momento mais adequado para a interrupção da gestação.^6^

Para o cardiologista, além da classificação correta da síndrome hipertensiva, é fundamental identificar sinais de gravidade e reconhecer gestantes com maior risco de desenvolver pré-eclâmpsia. A presença de comprometimento neurológico, renal, hepático ou cardiovascular indica progressão da doença e requer comunicação imediata com a equipe obstétrica, favorecendo uma abordagem multidisciplinar, integrada e oportuna.^7^

### Predição e prevenção da pré-eclâmpsia

A predição da pré-eclâmpsia evoluiu substancialmente na última década. Modelos combinados que integram características maternas, PA média, Doppler das artérias uterinas e biomarcadores angiogênicos, como o fator de crescimento placentário, alcançam acurácia de 75%-90% para a identificação, ainda no primeiro trimestre, de mulheres com risco de pré-eclâmpsia precoce ou pré-termo.^8^

As diretrizes contemporâneas recomendam de forma consistente a identificação precoce de fatores clínicos de alto risco, permitindo a implementação de medidas preventivas direcionadas. Entre elas, destacam-se o uso de ácido acetilsalicílico em baixa dose, preferencialmente iniciado antes de 16 semanas de gestação,^9^ a suplementação de cálcio em mulheres com baixa ingestão dietética e o incentivo a hábitos de vida saudáveis, incluindo prática regular de atividade física, alimentação equilibrada, controle do peso corporal e cessação do tabagismo (Figura Central).^10^

Entretanto, evidências recentes provenientes de revisões sistemáticas e metanálises demonstram heterogeneidade nos resultados relacionados à suplementação de cálcio, não sustentando benefício uniforme em todas as populações avaliadas.^11^

### Abordagem terapêutica durante a gestação

O tratamento da hipertensão na gestação deve ser instituído de forma oportuna e baseada em evidências. As diretrizes contemporâneas convergem para uma recomendação forte em favor do tratamento farmacológico de gestantes com PA ≥ 140/90 mmHg, particularmente quando as medidas não farmacológicas se mostram insuficientes para o controle da PA (Figura Central).^1–4^

Os estudos CHAP^5^ e CHIPS^12^ demonstraram que o tratamento da hipertensão leve a moderada reduz desfechos maternos adversos sem aumentar o risco de restrição do crescimento fetal. Esses achados sustentam as recomendações atualmente adotadas tanto pela Diretriz Brasileira de Hipertensão Arterial – 2025^1^ quanto pela *2025 ESC Guidelines for the Management of Cardiovascular Disease and Pregnancy*.^2^ As metas terapêuticas consistem em manter a PA sistólica entre 120 e 140 mmHg e a PA diastólica entre 80 e 90 mmHg, buscando equilíbrio entre a segurança materna e a preservação da perfusão útero-placentária.^1–4^

Na prática clínica, recomenda-se iniciar o tratamento com monoterapia, seguida de titulação gradual até a obtenção do controle da PA, respeitando-se as doses máximas seguras dos fármacos empregados. Trata-se de uma recomendação forte presente nas principais diretrizes internacionais. Para a metildopa, frequentemente são necessárias doses próximas de 2.000 mg/dia para obtenção de controle adequado, sendo considerada segura até 2.250 mg/dia. Para a nifedipina de liberação prolongada, doses eficazes costumam situar-se em torno de 90 mg/dia, com dose máxima de 120 mg/dia.^1–4,13^

No contexto brasileiro, deve-se considerar que a formulação de nifedipina de liberação prolongada de 30 mg não está disponível no Sistema Único de Saúde, aspecto que pode influenciar a estratégia terapêutica. A amlodipina pode ser considerada como alternativa quando a nifedipina não estiver disponível ou não for tolerada, embora as evidências que sustentam seu uso durante a gestação sejam menos robustas (Figura Central).

Quando o controle da PA não é alcançado com monoterapia, recomenda-se a associação de fármacos de primeira linha, preferencialmente metildopa combinada a um bloqueador dos canais de cálcio. Os betabloqueadores, especialmente o succinato de metoprolol, podem ser adicionados em etapa subsequente quando o controle permanecer inadequado. Essa estratégia escalonada reflete tanto a experiência clínica acumulada quanto a hierarquia das evidências disponíveis para o tratamento da hipertensão na gestação.

Por outro lado, os inibidores da enzima conversora da angiotensina (IECAs), os bloqueadores dos receptores da angiotensina II (BRAs), os antagonistas dos receptores mineralocorticoides e o atenolol são formalmente contraindicados durante a gestação, constituindo recomendação forte e consistente nas diretrizes contemporâneas.^1–4,13,14^

Embora as diretrizes internacionais priorizem o uso do labetalol, com base em metanálises^15,16^ que sugerem discreta superioridade em alguns desfechos maternos e perinatais, esse fármaco não está disponível no Brasil. Essa limitação, entretanto, não compromete a qualidade da assistência, considerando a eficácia e a segurança amplamente estabelecidas da metildopa e da nifedipina na prática clínica.

### Hipertensão grave e crise hipertensiva

A hipertensão grave constitui emergência obstétrica e requer tratamento imediato, mesmo na ausência inicial de lesão de órgão-alvo. A abordagem deve ser rápida, sistematizada e multidisciplinar, incluindo internação hospitalar, controle da PA com anti-hipertensivos de ação rápida (preferencialmente nifedipina por via oral ou hidralazina intravenosa) e investigação sistemática de comprometimento neurológico, renal, hepático ou cardiovascular.^1–4,6^

Em situações refratárias, a utilização de nitroglicerina intravenosa ou nitroprussiato de sódio pode ser considerada como medida temporária até a interrupção da gestação, sempre após avaliação criteriosa dos riscos e benefícios e no contexto de decisão multidisciplinar.^1^

O sulfato de magnésio constitui recomendação forte na presença de critérios de gravidade, sinais de iminência de eclâmpsia ou programação de parto, independentemente do anti-hipertensivo utilizado.^17^ Seu emprego requer monitorização clínica rigorosa, uma vez que a toxicidade pelo magnésio pode ocasionar complicações potencialmente graves, tornando essencial o reconhecimento precoce de seus sinais clínicos (Figura Central).

### Puerpério e risco imediato

Elevações pressóricas que surgem ou se agravam no puerpério devem ser consideradas como possível manifestação de pré-eclâmpsia até prova em contrário, justificando avaliação hospitalar, monitorização clínica e controle adequado da PA. Nessas situações, o sulfato de magnésio está indicado nos casos de hipertensão grave ou na presença de critérios de gravidade (Figura Central).

Evidências demonstram que o puerpério concentra parcela expressiva das complicações graves associadas às síndromes hipertensivas da gestação, incluindo eclâmpsia tardia, acidente vascular encefálico (AVE), edema agudo de pulmão e insuficiência cardíaca (IC).^6,7^ Muitos desses eventos ocorrem após a alta obstétrica e podem chegar inicialmente à avaliação cardiológica, reforçando a importância do reconhecimento precoce e da intervenção imediata.

Nessa fase, o tratamento farmacológico passa a seguir princípios mais próximos da terapêutica cardiovascular habitual. A metildopa, fármaco de primeira linha durante a gestação, deixa de ser a opção preferencial no puerpério em razão de seus efeitos centrais, incluindo sintomas depressivos, aspecto particularmente relevante nesse período. Embora estudos tenham demonstrado controle da PA semelhante entre a manutenção da metildopa e sua substituição por captopril no puerpério imediato, esses resultados derivam de seguimento de curto prazo e não contemplam desfechos neuropsiquiátricos tardios.^18^

Diferentemente do período gestacional, fármacos previamente contraindicados (p.ex., IECAs, os BRAs e os diuréticos) são compatíveis com a amamentação e devem ser considerados conforme as necessidades individuais da paciente, ampliando as opções terapêuticas disponíveis e favorecendo o adequado controle da PA.^7,14^

### Risco cardiovascular futuro: a gravidez como marcador precoce

A doença hipertensiva da gravidez deve ser reconhecida como um marcador precoce e independente de risco cardiovascular ao longo da vida, e não apenas como uma condição restrita ao ciclo gravídico-puerperal. Evidências consistentes demonstram que mulheres com história de pré-eclâmpsia ou hipertensão na gravidez apresentam risco aproximadamente duas a quatro vezes maior de desenvolver hipertensão arterial crônica, doença arterial coronariana, AVE e IC (particularmente IC com fração de ejeção preservada), muitas vezes já durante a primeira década após o parto.^19^

Essa associação transcende a simples observação epidemiológica e encontra respaldo em mecanismos fisiopatológicos bem estabelecidos, incluindo disfunção endotelial persistente, inflamação sistêmica crônica e alterações metabólicas associadas à pré-eclâmpsia.^20^

Nesse cenário, a doença hipertensiva da gravidez amplia o papel do cardiologista para além do manejo do evento agudo, incorporando a implementação de estratégias estruturadas de prevenção cardiovascular ao longo da vida. O registro sistemático do histórico obstétrico, a promoção de hábitos de vida saudáveis, o controle rigoroso dos fatores de risco cardiovasculares e o acompanhamento longitudinal constituem recomendações fortes da Diretriz Brasileira de Hipertensão Arterial – 2025,^1^ com potencial para modificar a trajetória clínica dessa população (Figura Central).

Ferramentas de estratificação de risco cardiovascular global, como o Systematic Coronary Risk Evaluation 2 e o Systematic Coronary Risk Evaluation 2 – Older Persons,^21^ recomendados pelas diretrizes europeias, bem como os modelos derivados das Pooled Cohort Equations,^22^ adotados pelas diretrizes norte-americanas, podem ser utilizados como instrumentos complementares na avaliação dessas mulheres. Embora tais escores não incorporem especificamente o histórico de doença hipertensiva da gravidez, essa condição deve ser reconhecida como um importante modificador de risco, justificando estratégias preventivas mais intensivas e acompanhamento clínico mais próximo.

## Conclusão

A doença hipertensiva da gravidez exige do cardiologista uma atuação integrada, multidisciplinar e contínua, que se estende desde o planejamento reprodutivo até o acompanhamento cardiovascular de longo prazo. Ao traduzir recomendações baseadas em evidências em condutas práticas e objetivas, esta carta científica, apoiada pela Figura Central, busca oferecer uma ferramenta de apoio à tomada de decisão em cenários nos quais o reconhecimento precoce, a precisão terapêutica e a integração assistencial são determinantes para os desfechos maternos.

Mais do que uma complicação obstétrica, a doença hipertensiva da gravidez representa uma oportunidade de identificação precoce de mulheres sob maior risco cardiovascular. Reconhecer essa condição e agir de forma proativa permite ampliar o impacto da cardiologia na promoção da saúde cardiovascular feminina ao longo de toda a vida.

## Data Availability

Os conteúdos subjacentes ao texto da pesquisa estão contidos no manuscrito.
