# Supplementary material for: Hipertensão na Gravidez: Das Diretrizes à Prática do Cardiologista
Source: Arq Bras Cardiol. 2026 Aug 14;123(8):e20260047. [Article in Portuguese] doi: 10.36660/abc.20260047 (PMC13671727; doi:10.36660/abc.20260047)
Supplement: Translation

# Translation: Hypertension in Pregnancy: From Guidelines to the Cardiologist's Clinical Practice

**DOI:** 10.36660/abc.20260047i

**Authors:** Walkiria Samuel Ávila

**Affiliations:** 1 São Paulo SP Brazil Instituto do Coração do Hospital das Clínicas da Faculdade de Medicina da Universidade de São Paulo, São Paulo, SP – Brazil

**Keywords:** Hipertension, Pregnancy, Pre-Eclampsia, Practice Guideline, Heart Disease Risk Factors

## Abstract

**Figure:** ACEI: angiotensin-converting enzyme inhibitor; APE: acute pulmonary edema; APS: antiphospholipid syndrome; ARB: angiotensin II receptor blocker; ASA: acetylsalicylic acid; BP: blood pressure; CAD: coronary artery disease; CKD: chronic kidney disease; ER: extended-release; HF: heart failure; HFpEF: HF with preserved ejection fraction; IR: immediate-release; IV: intravenous; PlGF: placental growth factor; PO: by mouth; SLE: systemic lupus erythematosus.

## Introduction

The Brazilian Guidelines of Hypertension– 2025^1^ of the Brazilian Society of Cardiology objectively address hypertension during pregnancy, dedicating a specific chapter to the topic. The document covers diagnostic definitions, strategies for the prediction and prevention of preeclampsia, therapeutic management during pregnancy and the postpartum period as well as the assessment of future cardiovascular risk associated with hypertensive disorders of pregnancy (HDPs).

In the international setting, the 2025 European Society of Cardiology (ESC) Guidelines for the Management of Cardiovascular Disease During Pregnancy provide detailed algorithms for proteinuria assessment, emergency management, and the treatment of hypertension and preeclampsia. Their recommendations are based predominantly on expert consensus, reflecting the scarcity of randomized clinical trials in this field.^2^

Although they adopt different methodologies — the Brazilian guideline based on the Grading of Recommendations Assessment, Development and Evaluation system and the European guideline structured around classes of recommendation and levels of evidence — both documents converge on key aspects of clinical practice. These include the recognition of blood pressure (BP) ≥ 140/90 mmHg as the diagnostic threshold for hypertension, the recommendation to treat mild-to-moderate forms of the disease, the need for immediate intervention in cases of severe hypertension, and the importance of ongoing cardiovascular follow-up for women with a history of HDPs. This convergence reflects the progressive alignment of major international guidelines following the publication of the 2021 International Society for the Study of Hypertension in Pregnancy recommendations, which standardized diagnostic criteria and BP measurement methods while reinforcing recommendations favoring active treatment of hypertension during pregnancy.^3,4^ These recommendations were subsequently strengthened by the results of the CHAP trial.^5^

Despite the growing harmonization of recommendations, the practical application of this knowledge remains a challenge for the general cardiologist. The classification of HDPs is not always intuitive, and common clinical situations (e.g., severe hypertension, postpartum BP increase, and superimposed preeclampsia) require early recognition and rapid decision-making. In this context, clinical synthesis tools may facilitate the implementation of current recommendations in routine practice. Central Illustration should be interpreted sequentially, following the numbered modules that summarize the key aspects of hypertension in pregnancy, from diagnostic definitions and risk stratification to clinical management and long-term cardiovascular implications.

### Definition, classification, and severity criteria

Hypertension in pregnancy is defined as the presence of BP ≥ 140/90 mmHg, which constitutes the common diagnostic threshold across the different forms of HDPs.^1–4^ The accurate distinction among these entities represents the first step toward a safe clinical approach, as it guides the intensity of clinical surveillance, the urgency of therapeutic intervention, and obstetric planning (Central Illustration).

In clinical practice, certain presentations constitute severe forms of HDPs and require immediate recognition. These include severe hypertension (systolic BP ≥ 160 mmHg and/or diastolic BP ≥ 110 mmHg), eclampsia (generalized tonic-clonic seizures), HELLP syndrome (hemolysis, increased liver enzymes, and thrombocytopenia), and posterior reversible encephalopathy syndrome, characterized by neurological manifestations associated with vasogenic edema predominantly affecting the parieto-occipital regions. These conditions reflect acute target-organ involvement and represent a major prognostic turning point, requiring immediate escalation of care, implementation of specific therapies, and assessment of the most appropriate timing for pregnancy termination.^6^

For cardiologists, in addition to correctly classifying the hypertensive syndrome, it is essential to identify severity features and recognize pregnant women at increased risk of developing preeclampsia. The presence of neurological, kidney, liver, or cardiovascular involvement indicates disease progression and requires immediate communication with the obstetric team, promoting a multidisciplinary, integrated, and timely approach.^7^

### Prediction and prevention of preeclampsia

The prediction of preeclampsia has evolved substantially over the past decade. Combined models integrating maternal characteristics, mean arterial pressure, uterine artery Doppler assessment, and angiogenic biomarkers, such as placental growth factor, achieve an accuracy of 75%-90% in identifying women at risk of early-onset or preterm preeclampsia during the first trimester.^8^

Contemporary guidelines consistently recommend the early identification of high-risk clinical factors, allowing the implementation of targeted preventive measures. These include the use of low-dose aspirin, preferably initiated before 16 weeks of gestation,^9^ calcium supplementation in women with low dietary calcium intake, and the promotion of healthy lifestyle habits, including regular physical activity, a balanced diet, weight control, and smoking cessation (Central Illustration).^10^

However, recent evidence from systematic reviews and meta-analyses has demonstrated heterogeneity in the results related to calcium supplementation, failing to support a uniform benefit across all evaluated populations.^11^

### Therapeutic approach during pregnancy

The treatment of hypertension during pregnancy should be initiated promptly and guided by evidence-based recommendations. Contemporary guidelines converge toward a strong recommendation in favor of pharmacological treatment for pregnant women with BP ≥ 140/90 mmHg, particularly when nonpharmacological measures prove insufficient to achieve adequate BP control (Central Illustration).^1–4^

The CHAP^5^ and CHIPS^12^ trials demonstrated that treatment of mild-to-moderate hypertension reduces adverse maternal outcomes without increasing the risk of fetal growth restriction. These findings support the recommendations currently adopted by both the Brazilian Guidelines of Hypertension – 2025^1^ and the 2025 ESC Guidelines for the Management of Cardiovascular Disease During Pregnancy.^2^ Therapeutic targets should maintain systolic BP between systolic BP between 120 and 140 mmHg and diastolic BP between 80-90 mmHg, seeking a balance between maternal safety and preservation of uteroplacental perfusion.^1–4^

In clinical practice, treatment should be initiated with monotherapy, followed by gradual dose titration until BP control is achieved, while respecting the maximum safe doses of the prescribed medications. This represents a strong recommendation included in the major international guidelines. For methyldopa, doses approaching 2,000 mg/day are often required to achieve adequate control, and the drug is considered safe up to 2,250 mg/day. For extended-release nifedipine, effective doses are typically around 90 mg/day, with a maximum recommended dose of 120 mg/day.^1–4,13^

In the Brazilian setting, it should be noted that the 30-mg extended-release nifedipine formulation is not available through the Brazilian Unified Health System, a factor that may influence therapeutic strategies. Amlodipine may be considered as an alternative when nifedipine is unavailable or not tolerated, although the evidence supporting its use during pregnancy is less robust (Central Illustration).

When adequate BP control is not achieved with monotherapy, the combination of first-line agents is recommended, preferably methyldopa combined with a calcium channel blocker. Beta-blockers, particularly metoprolol succinate, may be added in a subsequent step when BP control remains inadequate. This stepwise strategy reflects both accumulated clinical experience and the hierarchy of available evidence for the treatment of hypertension during pregnancy.

Conversely, angiotensin-converting enzyme inhibitors (ACEIs), angiotensin II receptor blockers (ARBs), mineralocorticoid receptor antagonists, and atenolol are formally contraindicated during pregnancy, constituting a strong, consistent recommendation across contemporary guidelines.^1–4,13,14^

Although international guidelines prioritize the use of labetalol on the basis of meta-analyses^15,16^ suggesting modest superiority in certain maternal and perinatal outcomes, this medication is not available in Brazil. This limitation however does not compromise the quality of care, given the well-established efficacy and safety of methyldopa and nifedipine in clinical practice.

### Severe hypertension and hypertensive emergencies

Severe hypertension constitutes an obstetric emergency and requires immediate treatment, even in the initial absence of target-organ damage. Management should be rapid, systematic, and multidisciplinary as well as include hospital admission, BP control with rapid-acting antihypertensive agents (preferably oral nifedipine or intravenous hydralazine), and systematic investigation of neurological, kidney, liver, or cardiovascular involvement.^1–4,6^

In refractory cases, the use of intravenous nitroglycerin or sodium nitroprusside may be considered as a temporary measure until delivery, always following careful assessment of risks and benefits and within the context of multidisciplinary decision-making.^1^

Magnesium sulfate carries a strong recommendation in the presence of severe features, signs of impending eclampsia, or planned delivery, regardless of the antihypertensive agent used.^17^ Its administration requires close clinical monitoring, as magnesium toxicity may lead to potentially serious complications, making the early recognition of its clinical manifestations essential (Central Illustration).

### Postpartum period and immediate risk

BP increases that emerge or worsen during the postpartum period should be considered a possible manifestation of preeclampsia until proven otherwise, warranting hospital evaluation, clinical monitoring, and adequate BP control. In these situations, magnesium sulfate is indicated in cases of severe hypertension or when severe features are present (Central Illustration).

Evidence demonstrates that the postpartum period accounts for a substantial proportion of severe complications associated with HDPs, including late-onset eclampsia, stroke, acute pulmonary edema, and heart failure (HF).^6,7^ Many of these events occur after obstetric discharge and may initially present to cardiology services, underscoring the importance of early recognition and prompt intervention.

At this stage, pharmacological treatment follows principles more closely aligned with conventional cardiovascular therapy. Methyldopa, a first-line agent during pregnancy, is no longer the preferred option in the postpartum period because of its central nervous system effects, including depressive symptoms, a particularly relevant consideration during this phase. Although studies have demonstrated similar BP control with either continuation of methyldopa or substitution with captopril in the immediate postpartum period, these findings derive from short-term follow-up and do not address long-term neuropsychiatric outcomes.^18^

Unlike during pregnancy, medications previously contraindicated (e.g., ACEIs, ARBs, and diuretics) are compatible with breastfeeding in most clinical scenarios and should be considered according to individual patient needs, thereby expanding available therapeutic options and facilitating adequate BP control.^7,14^

### Future cardiovascular risk: pregnancy as an early risk marker

HDPs should be recognized as an early and independent marker of lifelong cardiovascular risk rather than merely a condition confined to the pregnancy-postpartum period. Consistent evidence demonstrates that women with a history of preeclampsia or hypertension during pregnancy have an approximately two- to fourfold greater risk of developing chronic hypertension, coronary artery disease, stroke, and HF (particularly HF with preserved ejection fraction), often within the first decade after delivery.^19^

This association extends beyond a simple epidemiological observation and is supported by well-established pathophysiological mechanisms, including persistent endothelial dysfunction, chronic systemic inflammation, and metabolic alterations associated with preeclampsia.^20^

In this context, HDPs expand the role of the cardiologist beyond the management of the acute event, incorporating the implementation of structured lifelong cardiovascular prevention strategies. Systematic documentation of obstetric history, promotion of healthy lifestyle habits, rigorous control of cardiovascular risk factors, and longitudinal follow-up constitute strong recommendations of the Brazilian Guidelines of Hypertension – 2025,^1^ with the potential to modify the clinical trajectory of this population (Central Illustration).

Global cardiovascular risk assessment tools, such as the Systematic Coronary Risk Evaluation 2 and Systematic Coronary Risk Evaluation 2 – Older Persons,^21^ recommended by European guidelines, as well as models derived from the Pooled Cohort Equations,^22^ adopted by North American guidelines, may be used as complementary instruments in the evaluation of these women. Although these risk scores do not specifically incorporate a history of HDPs, this condition should be recognized as an important risk modifier, justifying more intensive preventive strategies and closer clinical follow-up.

## Conclusion

HDPs require an integrated, multidisciplinary, and continuous approach from the cardiologist, extending from reproductive planning to long-term cardiovascular follow-up. By translating evidence-based recommendations into practical and objective clinical guidance, this scientific letter, supported by Central Illustration, seeks to provide a decision-support tool for scenarios in which early recognition, therapeutic precision, and coordinated care are critical determinants of maternal outcomes.

More than an obstetric complication, HDPs represent an opportunity for the early identification of women at increased cardiovascular risk. Recognizing this condition and acting proactively may broaden the impact of cardiology on the promotion of women's cardiovascular health throughout the lifespan.

## Data Availability

The underlying content of the research text is contained within the manuscript.
